# Effect of emotional enhancement of memory on recollection process in young adults: the influence factors and neural mechanisms

**DOI:** 10.1007/s11682-018-9975-0

**Published:** 2018-10-25

**Authors:** Xiaoshu Li, Xiaohu Li, Shujuan Chen, Jiajia Zhu, Haibao Wang, Yanghua Tian, Yongqiang Yu

**Affiliations:** 1 Department of Radiology, The First Affiliated Hospital of Anhui Medical University, No.218 Jixi Road, Hefei, 230022 Anhui China; 2 Department of Neurology, The First Affiliated Hospital of Anhui Medical University, Hefei, China

**Keywords:** Emotional memory, Familiarity, Functional magnetic resonance imaging, Medial temporal lobe, Recollection

## Abstract

Emotional enhancement of memory (EEM) is thought to modulate memory recollection rather than familiarity. However, the contributing factors and neural mechanisms are not well understood. To address these issues, we investigated how valence, arousal, and the amount of devoted attention influence the EEM effect on recollection. We also compared the topological properties among hippocampus- and perirhinal and entorhinal cortex-mediated emotional memory processing networks. Finally, we evaluated the correlations between emotional memory/EEM and inherent properties (i.e., amplitude of low-frequency fluctuation and node degree, efficiency, and betweenness) of the hippocampus and perirhinal and entorhinal cortices in 59 healthy young adults by resting-state functional magnetic resonance imaging. EEM was elicited by incidental encoding, negative images, and positive high-arousal images. The hippocampus was correlated with recollection sensitivity and EEM_negative-high-arousal_. The emotional memory processing network mediated by the hippocampus had higher clustering coefficient, local efficiency, and normalized characteristic path length but lower normalized global efficiency than those mediated by the perirhinal and entorhinal cortices. The entorhinal cortex was associated with both recollection and familiarity sensitivity, but showed different correlation patterns. The perirhinal cortex was highly correlated with familiarity sensitivity of negative low-arousal stimuli. These results demonstrate that the EEM effect on memory recollection is influenced by valence, stimulus arousal, and amount of attention involved during encoding. Moreover, the hippocampus and perirhinal and entorhinal cortices play distinct roles in the recollection and familiarity of emotional memory and the EEM effect.

## Introduction

The dual-process theory of memory recognition states that recognition decisions are based on two processes—namely, recollection and familiarity (Wixted 2007; Yonelinas 2002). This theory is widely accepted by various investigators (Anderson et al. 2008; Besson et al. 2015; Evans and Wilding 2012; Wang et al. 2013). Recollection is a relatively slow process that consists of retrieving specific details associated with prior presentation of an item, whereas familiarity is a rapid process that acknowledges the fact that an item was previously encountered even though no contextual details are retrieved (Wixted 2007). The remember/know procedure is among the most widely used methods to separate recollection and familiarity from recognition. In this paradigm, subjects are required to introspect regarding the basis of their memory judgments and report whether they recognize items on the basis of remembering (i.e., recollection of episodic information about the study event) or knowing (i.e., the item is familiar in the absence of recollection) (Yonelinas 2002).

Emotional experiences can be described by two orthogonal dimensions: arousal (how calming or exciting) and valence (how negative or positive) (LaBar and Cabeza 2006), dividing emotional stimuli into five categories: negative with high arousal (NH), negative with low arousal (NL), neutral (N), positive with high arousal (PH), and positive with low arousal (PL). Emotional stimuli are more easily remembered than neutral stimuli, a phenomenon known as the emotional enhancement of memory (EEM) (Hamann 2001). Several behavioral studies have shown that EEM specifically modulates recollection rather than familiarity (Dolcos et al. 2005; Wang et al. 2013); however, they generally focused on valence while ignoring the effect of arousal on memory by simply categorizing stimuli as negative, neutral, and positive. In fact, amygdala and non-amygdala networks likely modulate memory for arousing and non-arousing emotional experiences, respectively (Kensinger 2004). The amygdala exerts a greater influence on the hippocampus and parahippocampal gyrus during the encoding of emotionally arousing as compared to neutral information. For non-arousing emotional events, memory may be boosted by differential engagement of prefrontal networks such as prefrontal-hippocampal interactions that control elaborative encoding (Savage et al. 2001). Additionally, the effect of arousal depends on stimulus valence (Mickley Steinmetz et al. 2010). Therefore, the conclusion that EEM specifically modulates the recollection process may be biased as it does not consider the role of arousal.

Two different mechanisms have been proposed for the EEM effect: one is associated with the effect of the amygdala on consolidation (i.e., the consolidation-mediation hypothesis) and the other is related to how the amygdala influences attention (i.e., attention–mediation hypothesis) (Hamann 2001; Hermans et al. 2014; Talmi et al. 2008). The latter is particularly relevant when retrieval occurs immediately after encoding, thereby eliminating the influence of consolidation. A previous study has shown that EEM in healthy young adults is due to different attention mechanisms activated during encoding: automatic processing (incidental) for negative stimuli and controlled processing (intentional) for positive stimuli (Sava et al. 2016). Intentional encoding refers to self-directed or controlled situations where there is an explicit attempt made and full attention devoted to encoding new episodic information. On the contrary, incidental encoding occurs under divided attention (Trivedi et al. 2011). For instance, young adults discriminated equally between old and new images in the full and divided attention condition; however, this pattern was altered in normal aging and neurodegenerative disease according to the attention resources available at encoding (Sava et al. 2016). Other studies have shown that dividing attention during encoding can disrupt memory enhancement for non-arousing emotional items but not emotionally arousing items for reasons that are not well understood (Kensinger 2004). It is also unknown whether attention-driven patterns of influence change when recollection and familiarity are separated from recognition retrieval.

Previous neuroimaging studies have demonstrated that recollection and familiarity have distinct underlying mechanisms. The perirhinal cortex is critical for memory familiarity; the hippocampus is important for recall or recollection (Montaldi and Mayes 2010; Wang et al. 2013; Yonelinas 2013); and the entorhinal cortex is thought to be associated with both recollection and familiarity (Dolcos et al. 2005). However, whether their functions are affected by different emotional states is unknown. Additionally, little is known about the correlations between these candidate brain regions and the unique EEM effect of emotional memory. Most previous studies have employed task-based functional magnetic resonance imaging (MRI), which directly detects brain activation. However, this method also has limitations such as the fact that only one domain and its related network are examined. In contrast, resting-state functional MRI overcomes limitations of task-based MRI by probing multiple neuronal networks and their inter-relationships simultaneously during a short period of acquisition (Barkhof et al. 2014). Additionally, resting-state functional MRI data are more easily acquired and can be applied to large populations, even those in which task-based functional MRI is not possible (Barkhof et al. 2014; Shen 2015). Diverse methods exist for analyzing resting-state functional MRI data (Chen and Glover 2015; Liu et al. 2017; Wang et al. 2016). Resting-state functional MRI has been successfully used to examine brain function in healthy subjects (Kiem et al. 2013; Sun et al. 2017) and clinical populations (Liu et al. 2013, 2015).

In the present study, we used resting-state functional MRI to examine the neural mechanisms of recollection and familiarity in emotional memory, with a focus on the correlations between the hippocampus and perirhinal and entorhinal cortices and EEM effects. The analytical methods used were the amplitude of low-frequency fluctuation (ALFF) and graph theory analysis. ALFF reflects spontaneous brain activity whereas the latter provides a mathematical framework for modeling the brain as a complex network or graph whose topological features (i.e., small-world property, network efficiency metrics, and regional measures) can be quantified (Xia and He 2017).

The objectives of the present study were to investigate: (1) how valence and arousal influence the EEM effect on recollection and familiarity; (2) how the manner of encoding (incidental or intentional) influence the EEM effect on recollection and familiarity; and (3) neural mechanisms of recollection and familiarity in emotional memory and the EEM effect. We hypothesized that the EEM effect on recollection was not only influenced by the intrinsic properties of emotional stimuli but also by the amount of attention allocated during the encoding phase, and that the hippocampus and perirhinal and entorhinal cortices play distinct roles in the recollection and familiarity of emotional memory.

## Materials and methods

### Participants

A total of 65 students at Anhui Medical University (Hefei, China) took part in this study. Four were excluded due to excessive head motion during MRI scans (translational or rotational motion parameters >2 mm or 2°); and two were excluded because of overall non-engagement in the experiment. Ultimately, 59 subjects were included in the analysis. Experimental procedures were approved by the Medical Research Ethics Committee of the First Affiliated Hospital of Anhui Medical University. Informed consent was provided by all subjects. The subjects were 18–30 years old; right-handed; cognitively normal; had no neurological or psychiatric disorders; were not taking psychoactive medication; had no addiction to alcohol or drugs; and were non-smokers. The characteristics of the study subjects are shown in Table 1.

**Table 1:** Demographics and cognitive features of participants

Items	Value
Number	59
Age(years,*x* ± *s*)	23.76 ± 2.52
Gender(male/female)	24/35
Education time(years,*x* ± *s*)	16.80 ± 2.30
Logical memory(immediate)	14.57 ± 3.78
Logical memory(30 min,delayed)	13.50 ± 4.52
Digit span (forward)	9.80 ± 1.17
Digit span (backward)	6.95 ± 1.31

### Emotional memory behavior test

#### Stimuli

The stimuli included 200 color photographs selected from the International Affective Picture System; these had two independent properties, each ranging from 1 to 9, with 1 corresponding to very negative on the valence scale and low emotional arousal on the arousal scale, and 9 corresponding to very positive on the valence scale and highly arousing on the arousal scale. The photographs were divided into two lists, each consisting of 100 stimuli (20 each of NH, NL, N, PH, and PL). The mean ratings were as follows: negative valence, ≤ 4.0; positive valence, ≥ 6.0; neutral valence, between 4.5 and 5.5; high arousal, ≥ 6.0; low arousal, ≤ 4.0; and neutral arousal, between 2 and 6. Each list was divided into two sets of 50 stimuli (10 each of NH, NL, N, PH, and PL); one set was used for the encoding phase, and all stimuli that appeared on the list were used during the retrieval phase. Thus, participants always saw 50 stimuli in the encoding phase and 100 in the retrieval phase (50 targets and 50 distractors).

#### Procedure

**Fig. 1:**
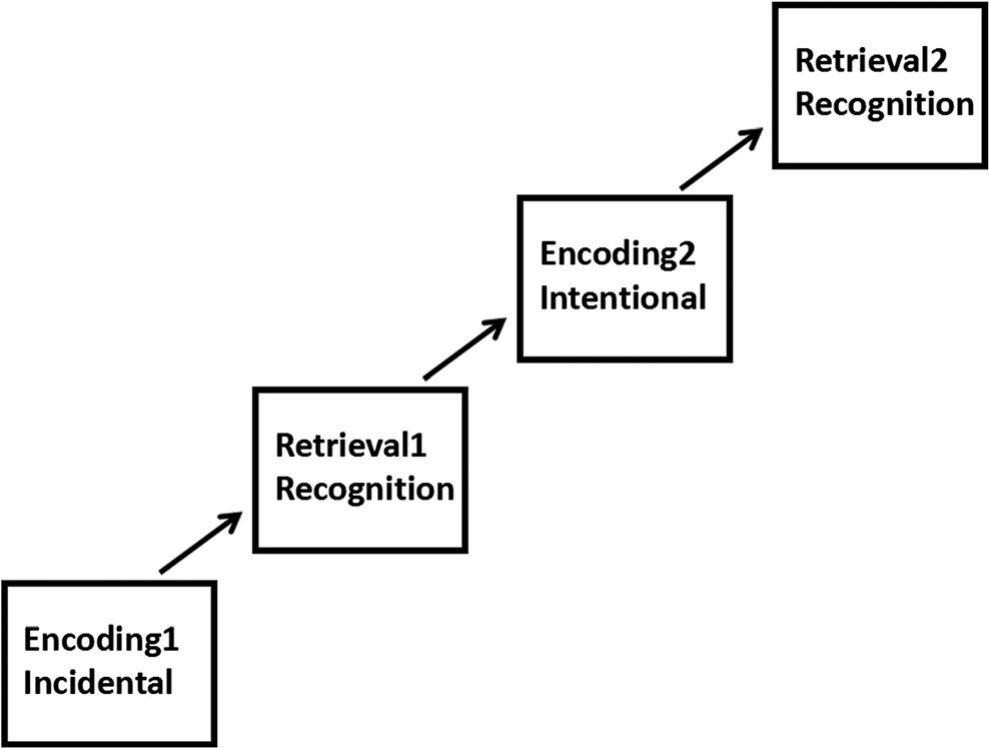
Encoding and retrieval steps. Step 1: incidental encoding and retrieval; step 2: intentional encoding and retrieval

The behavioral test was divided into two steps according to the manner of encoding. Each step had an encoding and retrieval phase. For the first step, participants were not informed of the subsequent retrieval task ahead of the encoding (incidental encoding). However, in the second step, they were informed of the recognition task and requested to encode as carefully as possible (intentional encoding) (Fig. 1). During encoding, participants were immediately asked to determine whether or not objects appearing in the photo were human (categorization task). The categorization task was used to ensure that participants visualized images in a valid way. The remember-know paradigm was employed in the retrieval phase, which was administered immediately after the encoding. Participants were first asked to make an old–new decision; for pictures considered “old”, they were subsequently required to introspect about the basis of their memory judgments and report whether they recognized items based on remembering or knowing. The experimenter was well trained to ensure that all the participants were given the same instructions. Before retrieval, participants were given a detailed explanation of the difference between “remember” and “know” responses. Some examples were provided to ensure that correct responses would be given in the experiment. After the test, participants were asked to describe the criteria they used for making judgments during the test so that the experimenter could verify the quality of the responses. By taking these measures, we tried to ensure that all participants could apply the same judgment criteria without confusing the concepts of recollection and familiarity. The tasks were performed on a laptop using E-prime v.2.0 software (PST Inc., Sharpsburg, PA, USA). During the encoding phase, a stimulus was displayed for 2000 ms with a 500-ms interval between stimuli.

## MRI scanning and data acquisition

MRI scans were performed on a General Electric 750w 3.0 T MRI scanner with a 24-channel head coil (General Electric, Waukesha, WI, USA). The imaging protocol included a T1-weighted three-dimensional structure sequence, resting-state blood oxygen level-dependent (BOLD) functional MRI sequence, axial T2-weighted and fluid-attenuated inversion recovery images. T1-weighted three-dimensional structural data were collected using the brain volume sequence [repetition time (TR)/echo time (TE)/inversion time = 8.5/3.2/450 ms; matrix = 256 × 256; field of view (FOV) = 256 mm × 256 mm; slice thickness = 1.0 mm without intervals); acquisition time was 4 min 56 s. BOLD data were collected using a gradient single-shot echo planar imaging sequence (TR/TE/flip angle = 2000 ms/30 ms/90°; matrix = 64 × 64; FOV = 220 mm × 220 mm; 185 volumes; 35 interleaved axial slices with slice thickness of 3 mm and interval space of 1 mm); acquisition time was 6 min 10 s.

## Resting-state functional MRI data preprocessing

BOLD data were preprocessed using Data Processing Assistant for Resting-state fMRI (DPARSF) software v.3.2 (http://rfmri.org/DPARSF). The processing steps included slice timing, motion correction, nuisance covariate regression, band-pass filtering, and normalization. The first 10 volumes for each participant were discarded to allow the signal to reach equilibrium and the participants to adapt to scanning noise. The remaining volumes were corrected for acquisition time delay between slices. Realignment was then carried out to correct the motion between time points. The frame-wise displacement (FD), which indexes volume-to-volume changes in head position, was also calculated. Nuisance covariates (six motion parameters, their first-time derivations, and white matter and cerebrospinal fluid signals) were regressed out from the data along with spike volumes whose FD exceeded 0.5, in order to minimize the influence of head movement. Datasets were then band-pass filtered in the frequency range of 0.01–0.08 Hz. Images were normalized to MNI space using the DARTEL technique, resampled into a 3-mm cubic voxel, and spatially smoothed using a 6-mm FWHM Gaussian kernel (Zhu et al. 2016).

## Network node selection and extraction

**Fig. 2:**
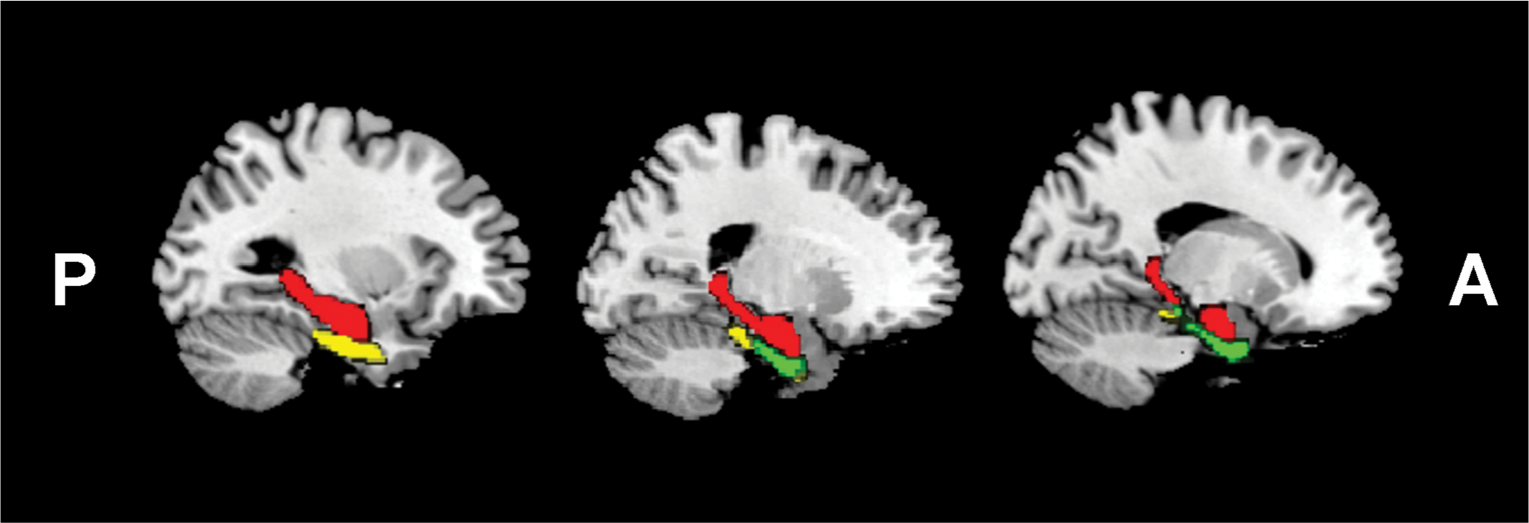
Regions of interests (unilateral). Red, hippocampus; green, entorhinal cortex; yellow, perirhinal cortex

The Human Brainnetome Atlas (Fan et al. 2016) was used to identify the following brain regions of interest (ROIs): bilateral hippocampus, perirhinal cortex, and entorhinal cortex (Fig. 2). Each pair along with 19 selected brain anatomical coordinates associated with emotion processing and regulation (Cisler et al. 2013) (Table 2) constituted the 21 nodes of the hippocampus-, perirhinal cortex-, and entorhinal cortex-mediated emotional memory processing networks. Time courses of each ROI were extracted and correlation matrices were calculated.

**Table 2:** Names and MNI coordinates of centroids of nodes associated with emotion processing and regulation

Node	MNI coordinate		
	X	Y	Z
Right amygdala	22	−2	−15
Left amygdala	−20	−4	−15
Right dorsolateral PFC	32	42	32
Left dorsal lateral PFC	−32	42	32
Right thalamus	10	−14	10
Left thalamus	−10	−14	10
Right caudate	14	12	12
Left caudate	−14	12	12
Subgenual cingulate	1	25	−11
Rostral anterior cingulate	2	36	6
Dorsal anterior cingulate	1	14	30
Medial PFC	1	60	6
Ventral medial PFC	1	51	−9
Right ventral lateral PFC	44	38	−8
Left ventral lateral PFC	−44	38	−8
Right anterior insula	38	20	0
Left anterior insula	−36	20	0
Right posterior insula	42	−12	2
Left posterior insula	−42	−12	2

*PFC* prefrontal cortex, *MNI* Montreal Neurological Institute

## Network analysis

GRETNA software (http://www.nitrc.org/projects/gretna) was used to perform whole-brain functional network analyses. We applied a range of sparsity thresholds—defined as the ratio of the number of existing edges divided by the maximum possible number of edges in a network—to all correlation matrices. A sparsity threshold range of 0.10 to 0.34 with an interval of 0.01 was used so that the generated networks were estimable for small-worldness and had sparse properties with the minimum number possible of spurious edges (Lei et al. 2015; Suo et al. 2015; Zhu et al. 2016).

Both global and regional network measures were calculated at each sparsity threshold for brain networks (Liu et al. 2016). Global measures included five small-world property and two network efficiency metrics. Small-world property metrics included the clustering coefficient Cp (a measure of the extent of local density or cliquishness of the network, which reflects network segregation); characteristic path length Lp (a measure of the extent of average connectivity or overall routing efficiency of the network, which reflects network integration); normalized clustering coefficient γ (ratio of clustering coefficients between real and random networks); normalized characteristic path length λ (ratio of the characteristic path lengths between real and random networks); and small-worldness property σ = γ/λ (scalar quantitative measurement of the small-worldness of a network). The number of random networks was set as 1000. Network efficiency metrics included the local efficiency Eloc (a measure of the fault tolerance of the network, reflecting how well the information is communicated within the neighbors of a given node when this node is eliminated) and global efficiency Eg (a measure of the global efficiency of parallel information transfer in the network). They were also scaled against mean Eloc and Eg obtained from 1000 matched random networks to obtain normalized Eloc (Eγ) and normalized Eg (Eλ). Regional measures included degree, betweenness, and efficiency. The degree of a node is the number of all edges for the node. The node betweenness is defined as the number of shortest paths between any two nodes that run through a given node. The nodal efficiency is the inverse of the harmonic mean of the minimum path length between a given node and all other nodes in the network (He et al. 2009a). The area under the curve was calculated for each network metric (He et al. 2009b; Zhu et al. 2016).

## Amplitude of low-frequency fluctuation (ALFF) calculation

ALFF is usually used to describe spontaneous brain activity in the resting state, providing another view of brain function. We calculated ALFF using DPARSF software v.3.2.

### Statistical analysis

#### Emotional memory behavior test

For each participant, we calculated the correct recognition rate of previously seen stimuli in the encoding phase (Hit) and false alarm rate (FA). The dual-process signal-detection/high-threshold model was used to explain the above recognition process. This model incorporates high-threshold theory and variance signal-detection model to explain recollection and familiarity processes, respectively (Yonelinas 1994; Yonelinas 2002). Recollection scores were calculated as Hit minus FA for “remember” responses (represented as “Pr” in this study). Familiarity scores were calculated based on the know response using an index of sensitivity (d’), with correction for independence (Yonelinas 2002). Since subjects were instructed to respond with “know” whenever an item was familiar and not recollected, the probability that an item was familiar was equal to the probability that it received a known response given that it was not recollected (Yonelinas 2002). A two-way analysis of variance (ANOVA) with repeated measures was performed on Pr and d’ respectively, with the inherent emotional dimension of the picture stimuli [valence (negative, neutral, positive) × arousal (high, low)] as the within-subject factor. The Bonferroni correction was used for post-hoc multiple comparisons.

#### Network graph analysis

The results of two-way ANOVA with repeated measures were evaluated for each global network metric (small-world properties and network efficiency), with network type as the within-subject factor. The Bonferroni correction was used for post-hoc multiple comparisons.

#### Correlation analysis

As mentioned previously, the Human Brainnetome Atlas was used to identify six ROIs: bilateral hippocampus, bilateral perirhinal cortex, and bilateral entorhinal cortex. Correlation analyses between each parameter (ALFF, and regional network metrics) extracted from the six ROIs and indices of emotional pictures [(i.e., Pr, d’, and EEM(Pr) (EEM = emotional − neutral)] were carried out using SPSS v.10.0 software (SPSS Inc., Chicago, IL, USA). Since only incidental encoding showed EEM effects of emotional stimuli, correlation analyses were performed only under the incidental condition.

## Results

### Emotional memory behavior test

**Fig. 3:**
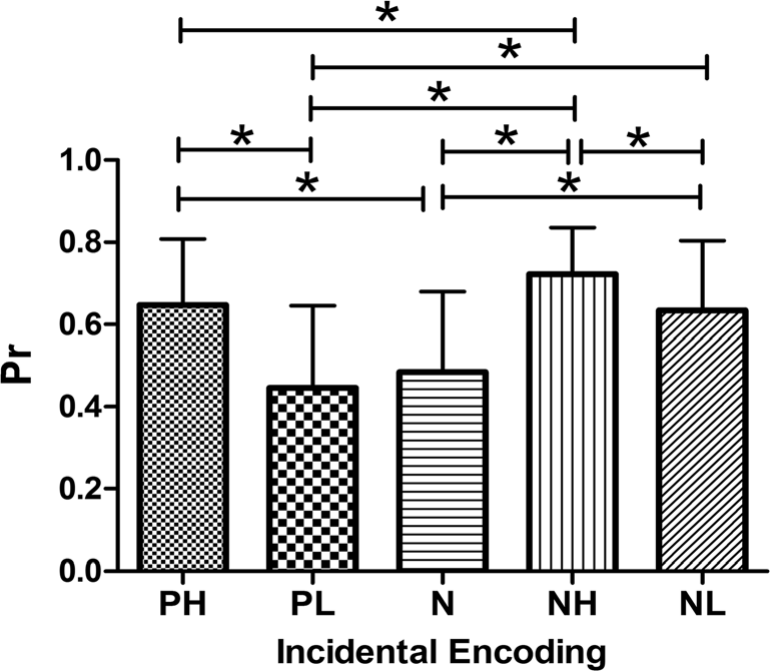
Recollection discrimination of incidental encoding. Negative pictures and positive high-arousal pictures showed a greater EEM effect than neutral ones. *P < 0.05 (pair-wise statistical difference, Bonferroni corrected). Bars represent standard deviation. Abbreviations: PH: positive with high arousal; PL: positive with low arousal; N: neutral; NH: negative with high arousal; NL: negative with low arousal

For incidental encoding, the index of recollection (Pr) showed significant main effects of emotion [F(4, 55) = 51.642, *P* < 0.001] (Fig. 3). Participants’ recollection was better for previously seen negative stimuli than for neutral ones (mean _*N*_ = 0.485), which was independent of arousal. That is, there was better discrimination for NH and NL than for neutral pictures (P < 0.001), and better recollection of NH than NL pictures (mean _NH_ = 0.722 and mean _NL_ = 0.634; *P* = 0.001). Thus, the arousal of negative picture stimuli determined discriminatory ability during recollection, but was not responsible for the EEM effect. However, this was not the case for positive stimuli, for which EEM effects still depended to some extent on arousal. There was better recollection of PH than of neutral pictures (mean _PH_ = 0.647, *P* < 0.001), but this did not apply to PL pictures. PL stimuli had no EEM effect on memory recollection compared to neutral stimuli (mean _PL_ = 0.447, *P* > 0.05). The difference between PH and NL was non-significant (P > 0.05). However, NH pictures were better recollected than PH pictures (*P* = 0.008). Nevertheless, for the familiarity index d’, there was no EEM effect at any level of emotional valence and arousal (*P* > 0.05 for all pairwise comparisons).

**Fig. 4:**
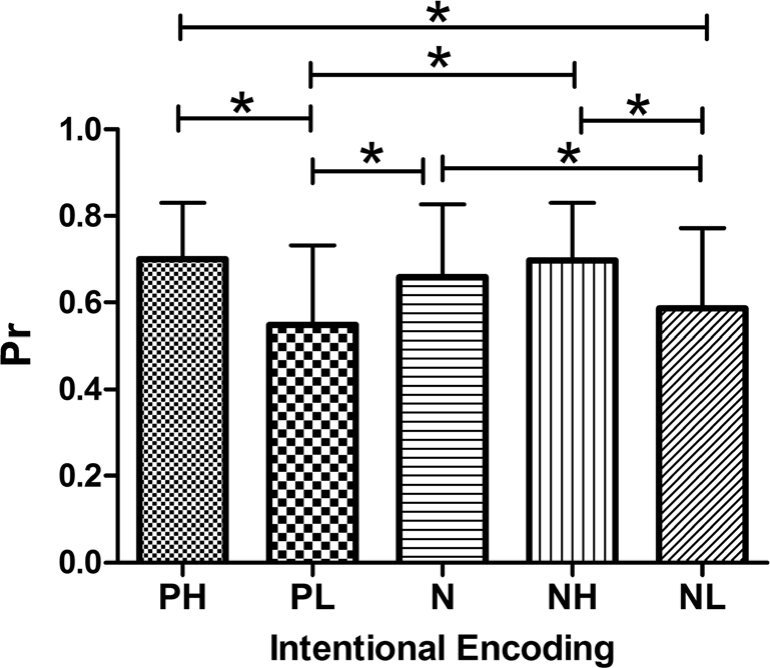
Recollection discrimination of intentional encoding. No EEM effect was observed relative to neutral pictures, which were better recollected than negative or positive pictures accompanied by low arousal. *P < 0.05 (pair-wise statistical difference, Bonferroni corrected). Bars represent standard deviation. Abbreviations: PH: positive with high arousal; PL: positive with low arousal; N: neutral; NH: negative with high arousal; NL: negative with low arousal

There was no EEM effect under the intentional encoding condition (Fig. 4). That is, there were no differences between the recollection sensitivity of NH/PH stimuli and that of neutral ones (mean _NH_ = 0.697, mean _PH_ = 0.700, and mean _*N*_ = 0.659; *P* > 0.05). Notably, there was better recollection of neutral than of NL or PL pictures (mean _NL_ = 0.587 and mean _PL_ = 0.548; *P* = 0.001 and *P* < 0.001, respectively). For the familiarity index d’, there was no EEM effect at any level of emotional valence and arousal (P > 0.05 for all pairwise comparisons).

### Network graph properties

**Fig. 5:**
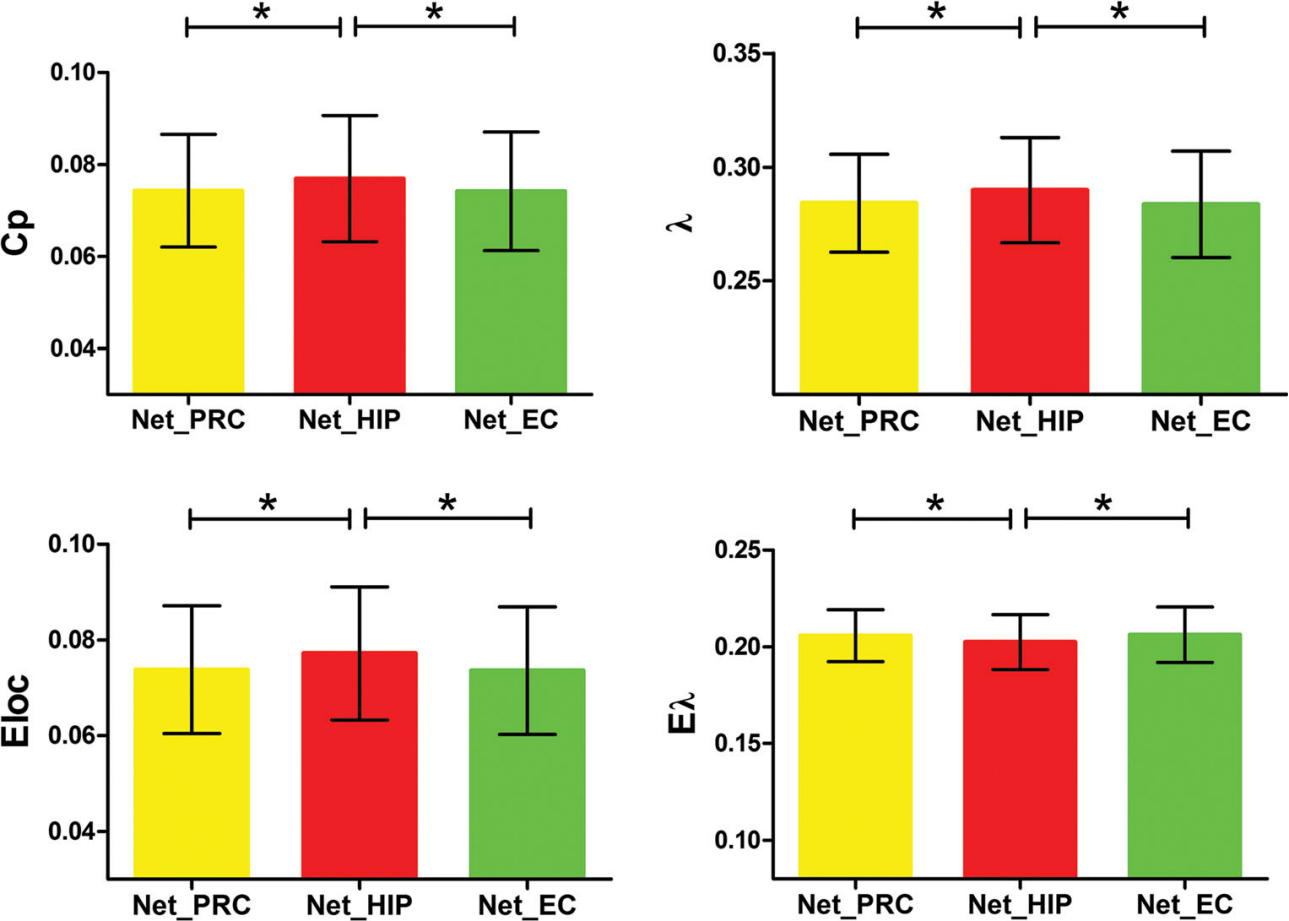
Network graph properties. The emotional memory network mediated by the hippocampus had higher Cp, Eloc, and λ but lower Eλ than those mediated by the perirhinal and entorhinal cortices. *P < 0.05 (pair-wise statistical difference, Bonferroni corrected). Bars represent standard deviation. Abbreviations: PRC: perirhinal cortex; HIP: hippocampus; EC: entorhinal cortex

For small-world properties, there were significant differences in Cp and λ among the three networks [F_Cp_(2, 57) = 4.765, *P* = 0.012; F_λ_(2, 57) = 5.666, *P* = 0.006]. Cp_hippocampus_ was greater than Cp_perirhinal cortex_ and Cp_entorhinal cortex_ (mean_hippocampus_ = 0.0770, mean_perirhinal cortex_ = 0.0743, mean_entorhinal cortex_ = 0.0742; *P* = 0.021 and 0.010, respectively); λ_hippocampus_ was greater than λ_perirhinal cortex_ and λ_entorhinal cortex_ (mean_hippocampus_ = 0.2899, mean_perirhinal cortex_ = 0.2841, mean_entorhinal cortex_ = 0.2836; P = 0.006 and 0.007, respectively) (Fig. 5). However, there were no differences in the other three small-world properties (i.e., Lp, γ, and σ) among the three networks.

For network efficiency properties, Eloc and Eλ differed significantly among the three networks [F_Eloc_(2, 57) = 11.901, *P* < 0.001; F_Eλ_(2, 57) = 7.193, *P* = 0.002]. Eloc_hippocampus_ was greater than Eloc_perirhinal cortex_ and Eloc_entorhinal cortex_ (mean_hippocampus_ = 0.0772, mean_perirhinal cortex_ = 0.0738, mean_entorhinal cortex_ = 0.0736; both P < 0.001), whereas Eλ_hippocampus_ was smaller than Eλ_perirhinal cortex_ and Eλ_entorhinal cortex_ (mean_hippocampus_ = 0.2025, mean_perirhinal cortex_ = 0.2059, mean_entorhinal cortex_ = 0.2063; *P* = 0.003 and P = 0.002, respectively) (Fig. 5). However, there were no differences in Eg and Eγ among the three networks.

### Correlation analysis

Regional network properties of the right hippocampus were positively correlated with Pr_NL_, Pr_PH_, Pr_PL_, and Pr_N_ (Table 3). Additionally, there were negative correlations between node degree of the right hippocampus and EEM_NH_ and between node efficiency of the same brain region and the same EEM effect (*r* = −0.348, *P* = 0.007 and *r* = −0.355, *P* = 0.006, respectively). Regional network properties of the entorhinal cortex were positively correlated with Pr_NL_ and Pr_PL_ (Table 3). The ALFF of bilateral entorhinal and left perirhinal cortices were positively correlated with d’_NL_. The node degree and node efficiency of left entorhinal cortex were negatively correlated with d’_PH_, while the node betweenness of the right entorhinal cortex was positively correlated with d’_NH_ (Table 4).

**Table 3:** Correlation analysis with recollection sensitivity of emotional memory

Brain Regions	Pr(NH) r/p	Pr(NL) r/p	Pr(N) r/p	Pr(PH) r/p	Pr(PL) r/p
Node Degree					
Right_HIP		0.322/0.013	0.389/0.002	0.355/0.006	0.381/0.003
Right_EC		0.274/0.036			
Node Efficency					
Right_HIP		0.265/0.042	0.340/0.008	0.320/0.014	0.352/0.006
Left_EC					0.264/0.043

*HIP* hippocampus, *EC* entorhinal cortex, *NH* negative with high arousal, *NL* negative with low arousal, *N* neutral, *PH* positive with high arousal, *PL* positive with low arousal

**Table 4:** Correlation analysis with familiarity sensitivity of emotional memory

Brain Regions	d’(NH) r/p	d’(NL) r/p	d’(N) r/p	d’(PH) r/p	d’(PL) r/p
ALFF					
Right_EC		0.279/0.032			
Left_EC		0.270/0.038			
Left_PRC		0.333/0.010			
Node Degree					
Left_EC				−0.282/0.031	
Node Efficiency					
Left_EC				−0.265/0.043	
Node Betweenness					
Right_EC	0.270/0.038				

*ALFF* amplitude of low-frequency fluctuation, *EC* entorhinal cortex, *PRC* perirhinal cortexm, *NH* negative with high arousal, *NL* negative with low arousal, *N* neutral, *PH* positive with high arousal, *PL* positive with low arousal

## Discussion

This study investigated the impact of valence and arousal of emotion and the amount of devoted attention during encoding on memory processes to examine the EEM effect on recollection or familiarity. Our results illustrate how emotions modulate brain functions during recollection and familiarity, and demonstrate for the first time that three emotional memory processing networks can provide new insight into this relationship.

Our results showed that the privileged EEM effect on recollection was influenced by attention during encoding. Only incidental encoding elicited the EEM effect on recollection. Interestingly, neutral pictures were better recollected than negative or positive pictures accompanied by low arousal under intentional encoding conditions. This phenomenon can be explained by the fact that participants may subjectively focus their attention on inherently unattractive neutral pictures. Some advanced cognitive and neural mechanisms such as those associated with attention and executive networks may play a role in intentional encoding, which will be the focus of a future investigation. This segregation of incidental and intentional encoding confirmed that the beneficial modulation of memory by emotions is an automatic and spontaneous process.

Our findings also confirmed that the EEM effect specifically modulates recollection rather than familiarity, which was consistent with previous studies (Dolcos et al. 2005; Wang et al. 2013). Recollection and familiarity are two types of retrieval that are differentially affected by emotional stimuli. Nevertheless, our findings indicate that under incidental encoding conditions, the EEM effect reflected in the recollection process was influenced by the inherent properties of emotional pictures, which was dependent not only on valence but also on arousal of emotional stimuli. For negative pictures, the EEM effect existed regardless of arousal; however, for positive pictures, the effect only appeared in association with high arousal. This segregation may be due to preferential processing of negative stimuli in young adults under conditions of limited attention. Young adults may spontaneously focus their attention on negative information and process it in a more self-referential way (Chainay et al. 2014); however, the opposite is true in older adults who preferentially process positive rather than negative stimuli (Kalenzaga et al. 2016; Kensinger and Schacter 2008; Leigland et al. 2004; Zhang et al. 2015). We therefore speculate that for young adults, valence rather than arousal predominantly contributes to the EEM effect of a negative picture; for a positive picture, high arousal may act in a complementary manner to generate the EEM effect. We also hypothesize that the pattern of the EEM effect on memory recollection—which is determined by intrinsic properties of emotional stimuli—may change with aging. That is, for old adults, the positive EEM effect may act on memory recollection regardless of arousal, whereas the negative EEM effect may act only under high-arousal conditions. However, further studies are needed to confirm this hypothesis.

We explored the relationships between critical brain regions associated with recollection/familiarity and the emotional memory/EEM effect. The hippocampus has long been considered as a key structure for episodic memory. However, as a component of the limbic system, there is increasing evidence for its role in emotion control (Catani et al. 2013; Fastenrath et al. 2014), as we reported in a previous study (Li et al. 2016). One mechanism that has been proposed for the EEM effect is the modulation of the hippocampus by the amygdala; another is the interaction between the hippocampus and prefrontal cortex (Savage et al. 2001). Our results showed that the Cp and Eloc of the hippocampus-mediated emotional memory processing and regulation network were greater than those of perirhinal cortex- and entorhinal cortex-mediated networks, indicating a higher functional segregation and fault tolerance. Functional segregation in the brain is the ability for specialized processing to occur within densely interconnected brain regions (Rubinov and Sporns 2010). Our findings imply that the hippocampus-mediated network plays a more specific and stable role in the emotional memory process. On the other hand, the greater λ and smaller Eλ of the hippocampus network indicates a longer path length and smaller parallel information transfer in the network, which correspond to lower functional integration. These complementary properties confer to the hippocampus-mediated network some of the properties of a regular network and reduce the information transfer speed. The hippocampus is thought to be highly correlated with memory recollection, which is a relatively slow memory recognition process (Montaldi and Mayes 2010; Wixted 2007). Our results support this possibility and highlight the neural mechanisms of recollection process at the network level.

We confirmed that the hippocampus was specifically correlated with the sensitivity of recollection rather than familiarity of emotional memory. Regional network properties (node degree and node efficiency) of the right hippocampus were negatively correlated with the EEM effect of recollection of negative high-arousal pictures. However, increases and decreases in BOLD signal should not be taken as evidence of increased and decreased involvement of a region in a particular computation—i.e., decreased BOLD signal could be due to increased precision (Leal et al. 2017). Thus, the negative correlation should not be interpreted as indicating that lower hippocampus function achieves better EEM effects; it should instead be considered as reflecting an association between the hippocampus and recollection sensitivity of emotional memory and the EEM effect. A previous study showed that negative high-arousal stimuli elicited positive connections between the amygdala and hippocampus, and stronger positive correlations among the amygdala, middle occipital gyrus, and fusiform gyrus (Mickley Steinmetz et al. 2010). However, these investigators did not focus on the EEM effect. Our results demonstrate that the hippocampus is involved in the EEM effect of the negative high-arousal modulation pathway. However, we failed to detect any correlations between the EEM effect of negative low-arousal pictures/positive pictures and the properties of the above-mentioned medial temporal lobe structures. One possible explanation is that we did not divide medial temporal lobe structures into sub-regions, which may limit the detection of potential correlations. Another explanation is that positive stimuli modulate the EEM effect through other pathways. For example, the prefrontal lobe may play an important role in the encoding of positive stimuli (Leigland et al. 2004).

The entorhinal cortex was correlated not only with recollection sensitivity of emotional pictures, but also with memory sensitivity of familiarity. Besides the amygdala and hippocampus, increased activity in the entorhinal cortex was induced by successful retrieval of emotional as compared to neutral pictures even after lengthy retention periods (Dolcos et al. 2005). Additionally, emotional arousal-enhanced successful encoding was found in the entorhinal cortex (Dolcos et al. 2004). These findings indicate that the entorhinal cortex is also associated with emotional memory and the EEM effect. Moreover, when recollection and familiarity were separated from recognition, the emotion effect was greater for recollection than for familiarity in the amygdala and hippocampus, whereas in the entorhinal cortex, it was similar for both forms of retrieval (Dolcos et al. 2005). Since the entorhinal cortex is an important node in the relay of information from the basolateral amygdala to hippocampus, it is expected to be associated with recollection sensitivity; it is also implicated in familiarity, underscoring the varied functions of this structure. Our findings were consistent with previous studies showing that the entorhinal cortex participates in emotional memory processing, and we confirmed that it is closely associated with both recollection and familiarity sensitivity. Notably, the correlation patterns of this structure differed in the two processes. The entorhinal cortex was positively correlated with recollection sensitivity of positive low-arousal images but negatively correlated with familiarity sensitivity of positive high-arousal pictures, implying that the it has distinct and segregated functions in recollection and familiarity.

We also found that the perirhinal cortex was correlated with the memory sensitivity of familiarity. The ALFF of the left perirhinal cortex was positively correlated with recognition sensitivity of the familiarity of negative low-arousal pictures. Data from human and animal studies indicate that the perirhinal cortex is implicated in familiarity discrimination for individual items and may in fact participate in an even broader range of memory functions, including associative and emotional memory as well as consolidation (Suzuki 1996). Our results demonstrate that the perirhinal cortex is closely correlated with familiarity sensitivity during emotional memory processing, especially for negative low-arousal emotional stimuli.

### Limitations

This study had some limitations. Firstly, the subjects included only young adults, which could limit the applicability of the conclusions to other age groups, and additional studies are needed to investigate potential changes during aging. Secondly, we did not separate medial temporal lobe structures into sub-regions, which may have precluded the detection of correlations with the EEM effect. Lastly, we did not examine the influence of sex in this study, although differences in emotional memory could exist between males and females. Further investigations focusing on the influence of sex and the underlying neural mechanisms are warranted.

## Conclusion

The findings of our study confirmed our primary hypothesis that the EEM effect specifically modulates recollection rather than familiarity, while being influenced by the intrinsic properties of emotional stimuli and the amount of attention allocated during encoding. The hippocampus-mediated network may play a highly specific and stable role in the emotional memory process. The longer path length and lower parallel information transfer speed of the hippocampus-mediated network reveals the neural mechanism underlying the recollection process at the network level. The entorhinal cortex is related to both recollection and familiarity sensitivity of emotional memory, but has different correlation patterns. The perirhinal cortex is correlated with memory sensitivity of familiarity. These results demonstrate that the hippocampus and perirhinal and entorhinal cortices play distinct roles in the recollection and familiarity sensitivity of emotional memory and the EEM effect in young adults.
